# The Effect of Patient Age and Sex on Decision-Making Around An Alzheimer’s Disease Blood Test: QUIP II Study Results

**DOI:** 10.1093/geroni/igaf122.2234

**Published:** 2025-12-31

**Authors:** Mark Monane, Kim Johnson, Daniel Parker, Robert Carlile, Darren Gitelman, Leslie Jacobs, Tim West, Joel Braunstein

**Affiliations:** C2N Diagnostics, St. Louis, Missouri, United States; Duke University, Durham, North Carolina, United States; Duke University, Durham, North Carolina, United States; Palmetto Primary Care Physicians, Summerville, South Carolina, United States; Advocate Health, Park Ridge, Illinois, United States; C2N Diagnostics, St. Louis, Missouri, United States; C2N Diagnostics, St. Louis, Missouri, United States; C2N Diagnostics, St. Louis, Missouri, United States

## Abstract

Blood biomarker (BBM) tests for Alzheimer’s disease represent accurate and accessible tools to aid healthcare providers (HCPs) in the evaluation of patients presenting with signs or symptoms of mild cognitive impairment or dementia. The objective of this analysis was to examine the effects of patient age and sex on clinical decisions using a commercially available mass spectrometry BBM test (PrecivityAD2™ test) yielding the Amyloid Probability Score 2 (APS2), which informs on the likelihood of a positive amyloid PET scan result. This secondary analysis of the QUIP II Study (NCT06025877) included 203 patients (average age 74 years, 53% female) evaluated by 12 HCPs representing 8 outpatient sites. Clinical decision-making was recorded by survey pre- and post-BBM testing. The composite primary endpoint, defined as a change in AD diagnostic certainty, AD medications, or additional brain amyloid evaluation pre- and post-BBM testing, was 75% (p < 0.0001 versus pre-specified threshold of 20% clinically meaningful change). The relationship between APS2 and composite endpoint was highly significant (p < 0.0001), yet was not significantly different when stratified by age (p = 0.94) or sex (p = 0.337). In a subgroup analysis of the three individual components of the composite endpoint, the only significant finding was that the magnitude of change in pre- and post-BBM AD diagnostic certainty was lower in the Negative APS2 patients aged 60-69 versus Negative APS2 patients aged 80 and older (p = 0.01), likely related to a lower pre-test AD probability among younger patients. These study results support the usefulness and generalizability of this BBM test in clinical care.

